# Phenotypic and Gene Expression Alterations in Aquatic Organisms Exposed to Microplastics

**DOI:** 10.3390/ijms26031080

**Published:** 2025-01-26

**Authors:** Yun Ju Lee, Woo Ryung Kim, Eun Gyung Park, Du Hyeong Lee, Jung-min Kim, Hyeon-su Jeong, Hyun-Young Roh, Yung Hyun Choi, Vaibhav Srivastava, Anshuman Mishra, Heui-Soo Kim

**Affiliations:** 1 Department of Integrated Biological Sciences, Pusan National University, Busan 46241, Republic of Koreajmk95@naver.com (J.-m.K.); tbd97@pusan.ac.kr (H.-s.J.);; 2 Institute of Systems Biology, Pusan National University, Busan 46241, Republic of Korea; 3 Department of Biochemistry, College of Korean Medicine, Dong-Eui University, Busan 47227, Republic of Korea; 4 Division of Glycoscience, Department of Chemistry, School of Engineering Sciences in Chemistry, Biotechnology and Health, KTH Royal Institute of Technology, AlbaNova University Center, 106 91 Stockholm, Sweden; vasri@kth.se; 5 Institute of Advanced Materials, IAAM, Gammalkilsvägen 18, 590 53 Ulrika, Sweden; 6 International Institute of Water, Air Force Radar Road, Bijolai, Jodhpur 342003, India; 7 Department of Biological Sciences, College of Natural Sciences, Pusan National University, Busan 46241, Republic of Korea

**Keywords:** aquatic organism, gene expression, heavy metal, microplastic, organic compound, phenotype, pollutant, qPCR, sequencing, water

## Abstract

The use of plastics, valued for its affordability, durability, and convenience, has grown significantly with the advancement of industry. Paradoxically, these very properties of plastics have also led to significant environmental challenges. Plastics are highly resistant to decomposition, resulting in their accumulation on land, where they eventually enter aquatic environments, due to natural processes or human activities. Among these plastics, microplastics, which are tiny plastic particles, are particularly concerning when they enter aquatic ecosystems, including rivers and seas. Their small size makes them easily ingestible by aquatic organisms, either by mistake or through natural feeding behaviors, which poses serious risks. Moreover, microplastics readily adsorb other pollutants present in aquatic environments, creating pollutant complexes that can have a synergistic impact, magnifying their harmful effects compared to microplastics or pollutants acting alone. As a result, extensive research has focused on understanding the effects of microplastics on aquatic organisms. Numerous studies have demonstrated that aquatic organisms exposed to microplastics, either alone or in combination with other pollutants, exhibit abnormal hatching, development, and growth. Additionally, many genes, particularly those associated with the antioxidant system, display abnormal expression patterns in these conditions. In this review, we examine these impacts, by discussing specific studies that explore changes in phenotype and gene expression in aquatic organisms exposed to microplastics, both independently and in combination with adsorbed pollutants.

## 1. Introduction

Plastics are indispensable materials used in industries worldwide, due to their versatile properties, such as low cost, lightweight, excellent durability, moldability, and biological inertness [1,2,3,4]. Consequently, global plastic production increases annually, reaching a staggering 459.75 million tons in 2019 alone [5]. However, as plastic consumption grows, these materials increasingly accumulate in terrestrial environments and are subsequently transported into aquatic ecosystems through natural processes, such as wind and stormwater runoff [6,7,8,9]. Moreover, human activities, such as fishing, recreational pursuits, and improper waste disposal, contribute to the influx of plastics into aquatic systems [10,11,12]. The accumulation of plastics in aquatic ecosystems increased consistently from 2000 to 2019, reaching a total of 139.85 million tons by 2019, with 30.41 million tons deposited in oceans and 109.44 million tons in rivers and lakes [13].

Among the plastics entering aquatic environments, microplastics, small plastic particles smaller than 5 mm in size, are a cause for particular concern due to their ubiquity and ecological implications [14,15,16,17]. Microplastics are broadly classified into two categories: primary microplastics, which are intentionally manufactured as small particles (e.g., microbeads in cosmetics), and secondary microplastics, which result from the fragmentation of larger plastic items through physical, chemical, and biological processes [18,19,20,21,22,23]. Both types of microplastics are pervasive in aquatic systems and are readily ingested by aquatic organisms, due to their small size. Microplastics are detected in aquatic species, such as fish and seaweed, and their accumulation is analyzed using techniques such as density separation, microscopy, and Fourier transform infrared spectroscopy [24,25,26].

The ingestion of microplastics by aquatic organisms has been linked to a variety of biological effects, including those impacting growth, development, and embryonic hatching times, as well as modifications in the expression of genes related to stress or developmental processes [27,28,29,30]. Furthermore, microplastics display diverse characteristics in aquatic environments, such as variations in concentration, particle shape, and type, and exhibit a high capacity to adsorb other pollutants, including heavy metals [28,31,32,33]. Consequently, ongoing research is being carried out that examines the effects of microplastics on aquatic life, considering these various parameters. Therefore, in this review, we aim to explore the impacts of microplastics on the phenotype and gene expression of aquatic organisms subject to various microplastic conditions, using specific examples.

## 2. The Impact of Microplastics on Aquatic Organisms

With increasing awareness of the detrimental effects of microplastic pollution, research into its effects on aquatic organisms has intensified [19,34]. Numerous studies have reported the accumulation of microplastics in various tissues from aquatic species, raising significant concerns about ingestion and subsequent biological effects [35]. Evidence suggests that microplastics can induce physiological changes in aquatic organisms, particularly by affecting phenotypic traits and altering gene expression [36,37,38]. To further elucidate these effects, researchers have investigated the effects of varying microplastic conditions, such as concentration and particle size, in different species [39,40]. This section summarizes the impacts of microplastics on the phenotypic characteristics and gene expression patterns of aquatic organisms subject to various environmental conditions.

### 2.1. Phenotypic Changes in Aquatic Organisms Following Microplastic Ingestion

Microplastic ingestion has been shown to induce a wide range of phenotypic changes in aquatic organisms, affecting their growth, development, reproduction, and overall health (Table 1). For instance, *Sebastes schlegelii* exposed to microplastics exhibited longer feeding times and significantly reduced foraging time and swimming speeds than the non-exposed groups [41]. Additionally, microplastic-exposed *Sebastes schlegelii* displayed restricted movement, remaining active within less than half the tank area compared to the untreated fish. These behavioral changes suggest that microplastic exposure impairs their hunting and exploratory capabilities, potentially increasing predation risk. To better understand the impact of microplastics on aquatic ecosystems, studies have explored the effects of different microplastic types and exposure conditions across various species. For example, in *Carassius auratus*, the ingestion of microplastic fragments caused significant damage to the buccal epithelium, including exfoliation, abrasions, and severe dermal damage to the lower jaw [42]. Degenerative changes were observed in both the upper and lower jaws, with more severe damage caused by fragments than pellets. Similarly, exposure to virgin and harbor microplastics affected the growth and survival of *Ambassis dussumieri* [29]. Both groups showed reduced length and body depth compared to the controls, with survival rates declining after 50 days of exposure, highlighting the long-term impact of microplastics on fish health.

Microplastics also impact non-fish taxa, such as echinoderms. High concentrations of microplastics caused developmental arrests and malformations in *Sphaerechinus granularis* embryos, particularly during the blastula–gastrula phase [43,44]. Increased mitotic abnormalities and larval malformations were observed, confirming that microplastic exposure has toxic effects on early development. Similarly, detrimental effects have been reported in crustaceans, such as *Ceriodaphnia dubia* [45]. Exposure to two types of microplastics, namely fibers and beads, decreased organism survival rates, with fibers exerting a more pronounced effect. While organism reproduction was unaffected at low fiber concentrations, it sharply declined at higher levels. In regard to both types of microplastics, increasing the microplastic concentration resulted in the reduced body size of the organism, indicating a dose-dependent impact on fitness. These findings demonstrate that while the degree of and type of microplastic impact varies, their presence consistently exerts negative effects across a range of aquatic taxa, from behavioral and developmental disruptions to survival and reproduction impairments.

**Table 1:** Microplastic exposure conditions and resulting phenotypic changes in aquatic organisms. NA: Not Available.

Microplastic Type	Microplastic Size	Microplastic Concentration	Exposure Time	Phenotype	Experiment Model	Reference
polystyrene	15 μm	1 × 10^6^/L	14, 21 days	feeding time, foraging time, swimming speed, histopathological changes	*Sebastes schlegelii*	[41]
polystyrene, polyethylene acrylate	fiber: 0.7–5.0 mm fragment: 2.5–3.0 mm pellet: 4.9–5.0 mm	fiber: 55–76 particles fragment: 15 particles pellet: 15 particles	6 weeks	buccal cavity, jaw structure	*Carassius auratus*	[42]
polyethylene, polyvinyl chloride, polystyrene	250–1000 μm	0.05 g	10 min, 24, 48, 72, 96 h	length, body depth, mass, survival probability	*Ambassis dussumieri*	[32]
powdered plastics	100 μm	10, 20, 40, 60, 80, 100, 200, 250 mg/L	24, 96 h	antioxidant system, photosynthetic activity, growth	*Chlorella vulgaris*	[46]
polystyrene	1 μm	0.85, 8.5, 85, 850, 8500 μg/L	24, 48 h, 6, 7 days	offspring reproduction/growth inhibition, oxidative stress	*Brachionus calyciflorus*, *Ceriodaphnia dubia*, *Heterocypris incongruens*	[47]
polypropylene, low-density polyethylene	125–1000 μm	0.75, 8.25 μg/L	21 days	micronucleated erythrocytes	*Cyprinus carpio*	[48]
polystyrene	3 μm	0.05, 0.25, 1.25, 6 mg/L	7 days	photosynthetic pigments, oxidative stress, antioxidant system	*Egeria densa*	[49]
polypropylene	11.86–44.62 μm	250, 500, 750 mg/g	28 days	oxidative stress, antioxidant system, digestive system, histopathological changes	*Pomacea paludosa*	[50]
polyester, polyethylene	100–400 μm	31.3, 62.5, 125 L, 250, 500, 1000, 2000 μg/L, 4 mg/L	48 h, 8 days	mortality, reproductive output, body size	*Ceriodaphnia dubia*	[45]
polystyrene, polymethyl methacrylate	10, 50, 80, 230 μm	0.1, 1, 5, 50 mg/L	10–72 min post-fertilization	embryo development, fertilized eggs, offspring developmental defects	*Sphaerechinus granularis*	[43]
polypropylene	11.86–44.62 μm	100, 500, 1000 mg/kg of dry food	96 h, 14 days	antioxidant system, oxidative stress, histopathological changes, transmission of nerve impulses	*Oreochromis mossambicus*	[51]
polypropylene	10–27 μm	10, 22.5, 45, 90, 100, 1000, 5000, 10,000, 20,000 microplastics/mL	0, 10, 28, 42 days	mortality, dry weight, egestion time, number of neonates	*Hyalella azteca*	[52]
polyethylene	<400 μm	0.01, 0.02, 0.04, 0.08 g/mL	30, 60 min	feeding rates, morphology, hydranth numbers	*Hydra attenuata*	[53]
polypropylene, polyvinyl chloride	<236 μm	5, 10, 50, 100, 250, 500 mg/L	1–11 days	photosynthetic pigments, photosynthetic activity, rapid light-response curves	*Chlorella pyrenoidosa*, *Microcystis flos-aquae*	[54]
polystyrene	300–600 nm	5, 25, 50, 100 mg/L	12, 24 h, 1–10 days	growth, photosynthetic pigments, photosynthetic activity, lipid peroxidation	*Chlamydomonas reinhardtii*	[55]
polystyrene	50 μm	10, 10^3^, 10^5^ particles/L	24, 48, 72 h	growth, antioxidant system, photosynthetic activity	*Chlorella marine*, *Nannochloropsis oculata*, *Phaeodactylum tricornutum*, *Chlorella vulgaris*, *Tetradesmus obliquus*	[56]
polystyrene	1, 12 μm	0.1, 1, 10 mg/L	NA	viability, oxidative stress, antioxidant system, photosynthetic activity, metabolic activity, lipid peroxidation, membrane integrity	*Scenedesmus obliquus*	[57]

### 2.2. Impact of Microplastic Ingestion on Gene Expression and Function in Aquatic Organisms

In the previous section, we discussed the altered phenotypes of aquatic organisms exposed to microplastics. To further understand these changes, it is essential to investigate gene expression, as variations in gene activity underlie many cellular functions and potentially drive phenotypic changes [58,59,60,61]. Gene expression can be analyzed using two primary methodologies: RNA sequencing and quantitative polymerase chain reaction (qPCR). RNA sequencing provides a comprehensive overview of all the expressed genes, whereas qPCR is a targeted approach, designed to quantify the expression levels of specific genes [61,62,63]. The choice of methodology depends on the research objectives and this section is organized to reflect these distinctions.

#### 2.2.1. Functional Implications of Differentially Expressed Genes in Aquatic Organisms Exposed to Microplastics

To investigate the effects of microplastics on cellular functions, RNA sequencing is frequently employed to identify differentially expressed genes, which are further analyzed through Gene Ontology (GO) or Kyoto Encyclopedia of Genes and Genomes (KEGG) analyses (Table 2). GO analysis identifies associated biological functions and KEGG analysis explores the metabolic pathways affected. For example, in *Penaeus vannamei*, RNA sequencing revealed 1251 differentially expressed genes, with 684 upregulated and 566 downregulated after microplastic exposure [64]. GO enrichment analysis showed that upregulated genes were involved in the adaptation of rhodopsin-mediated signaling and metarhodopsin inactivation, whereas downregulated genes were involved in adult somatic muscle development and muscle organ development. KEGG pathway analysis highlighted that 515 of these genes were linked to abnormalities in metabolic pathways, including calcium channels critical for cardiac muscle contraction, suggesting that cardiac dysfunction was induced by microplastics. Similarly, in *Mytilus galloprovincialis*, RNA sequencing identified differentially expressed genes following its exposure to spherical and fiber-shaped microplastics over 4 and 14 days [31]. The exposure to spherical microplastics for 4 and 14 days led to 142 common differentially expressed genes associated with metabolic processes and membrane-based cellular components, while the exposure to fiber-shaped microplastics resulted in 157 genes linked primarily to metabolic regulation. GO analysis showed distinct differences, namely that spherical microplastics were associated with cellular components, and fiber-shaped microplastics were related to metabolic processes. Clustering analysis further revealed that microplastic-exposed mussels exhibited stress responses, apoptosis regulation, and antioxidant mechanisms, highlighting diverse cellular impacts, based on the shape of the microplastics and the duration of exposure. Another study explored the effects of microplastics on *Euglena gracilis,* based on the size and condition of the microplastics [65]. The exposure to large microplastics caused a concentration-dependent increase in growth inhibition, which plateaued beyond a certain threshold. In contrast, small microplastics showed a consistent increase in growth inhibition with an increase in the microplastic concentration and demonstrated greater toxicity than large microplastics at higher concentrations. Sequencing analysis identified 43 differentially expressed genes in response to small microplastics and 47 differentially expressed genes in response to large microplastics. KEGG pathway enrichment analysis revealed that small microplastics induced the downregulation of cellular processes and environmental information processing, whereas large microplastics inhibited carbohydrate metabolism and signal transduction pathways. These findings suggest that microplastics’ toxicity mechanisms vary according to the size of the microplastic, affecting the growth of aquatic organisms via distinct molecular pathways.

#### 2.2.2. Gene Expression Changes in Aquatic Organisms Following Microplastic Exposure

Some researchers have observed microplastic effects in aquatic organisms by examining specific gene expression changes caused by microplastic exposure across various species without performing transcriptome sequencing (Table 3) [32,69]. To better understand these impacts, many studies have examined both phenotypic and gene expression changes. For instance, one investigation focused on the effects of primary and secondary microplastics on *Oryzias melastigma* embryos [28]. Exposure to both types of microplastics shortened their hatching time without affecting embryo mortality or hatching rates, but no changes were observed in hatching-related gene expression. However, the average oxygen influx and embryo development were adversely affected. Specifically, primary microplastic exposure reduced the average oxygen influx, while secondary microplastic exposure increased it. Both microplastic types upregulated *HIF-1α*, a hypoxia-related gene. Secondary microplastics also upregulated *GATA4* and *NKX2.5*, genes associated with heart development, while downregulating *COX2*, a gene linked to cardiovascular inflammation. Although the study did not identify specific genes influencing hatching times, it did uncover genes associated with oxygen uptake and cardiovascular changes, highlighting the greater negative impact of secondary microplastics compared to primary microplastics. Another study explored microplastic exposure effects on *Oryzias melastigma* over 60 days, revealing concentration-dependent microplastic accumulation in the liver [32]. Gene expression analysis showed decreased levels of *vitellogenin 1*, *vitellogenin 2*, *ChgH*, *ChgL*, and *ERα* in female livers. Exposed fish also exhibited reduced egg production, lower offspring fertility, decreased hatching rates, slower embryo heart rates, and shorter offspring body lengths. These findings indicate that microplastic exposure inhibits vitellogenin and choriogenin synthesis, delays ovarian development, and adversely affects reproduction in marine medaka. In another study, microplastic-induced physiological changes were investigated in zebrafish [39]. The microplastic exposure led to the generation of reactive oxygen species (ROS) and lipid peroxidation, with the effects varying based on the exposure time and concentration of the microplastics. Antioxidant biomarkers, such as CAT, SOD, and GPx, exhibited suppressed activity, whereas the GST activity increased. Gene expression analysis revealed that higher microplastic concentrations downregulated *CAT*, *SOD1*, *gpx1a*, and *ACHE* genes, while upregulating *gstp1*, *hsp70*, and *ptgs2a*. These findings demonstrate that microplastics induce ROS-mediated stress responses in zebrafish in a concentration- and time-dependent manner. A study on *Chlorella pyrenoidosa* examined the influence of the size and concentration of microplastics [70]. The cumulative growth rates significantly declined in groups exposed to small microplastics compared to the control, along with reductions in photosynthetic pigments, such as chlorophyll a, chlorophyll b, and carotenoids. Large microplastics had a lesser impact, while higher concentrations led to more pronounced impacts. Significant differences were observed in regard to extracellular polymeric substances, soluble proteins, and MDA levels, particularly in groups exposed to small microplastics at high concentrations. Gene expression analysis showed the increased expression of *rbcS* and *chlL*, photosynthesis-related genes, for up to 5 days in high-concentration small-microplastic groups. The expression of *ATPF1B*, an energy metabolism-related gene, also increased over time. Similarly, low small-microplastic concentrations elevated *rbcS*, *rbcL*, and *ATPF1B* expression, with levels remaining higher than the control, with small microplastics exerting a greater impact than larger ones.

#### 2.2.3. Functional Analysis and Gene Expression Influenced by Microplastics in Aquatic Organisms

Several studies have employed RNA sequencing to investigate the impacts of microplastic ingestion on marine organisms, with subsequent gene expression validation using qPCR (Table 4). For instance, the RNA sequencing of liver tissues from *Danio rerio* exposed to two different concentrations of microplastics revealed alterations in the expression of genes associated with key biological processes [82]. In both the high- and low-concentration exposure groups, the expression levels of *ltb4r* and *ifitm1* (immune response-related genes), as well as *elovl6* and *ch25h* (lipid metabolism-related genes), were found to be reduced. These findings suggest that microplastic exposure influences immune and metabolic pathways in the liver, potentially impairing its function. In another study, *Artemia salina* was exposed to an artificial seawater medium, containing four concentrations of microplastics, to evaluate their effects [83]. While the survival rates were unaffected, microplastics accumulated in a concentration-dependent manner, inducing ROS generation. Whole transcriptome sequencing identified 721 differentially expressed genes in response to microplastic exposure, with many associated with apoptosis and ROS-related pathways. Among these, seven genes related to immune response, oxidative stress, and apoptosis, such as *early growth response protein 1b*, *titin*, *MHC class 1 antigen*, *Crammer*, *Pyrimidodiazepine synthase*, *Dappudraft_310496*, and *Dappudraft_308348*, showed increased expression in the microplastic-treated groups compared to the controls. These results confirm the molecular-level toxicity of microplastics. Additionally, the life history traits of *Oryzias melastigma* exposed to varying concentrations of microplastics were examined [84]. Independent of the microplastic concentration, reductions were observed in the hatching rate, body length, body weight, and heartbeat of the organisms. Males exposed to concentrations exceeding 20 μg/L exhibited a decreased body length and gonadosomatic index. Similarly, females exhibited reductions in body length, weight, and gonadosomatic index. Furthermore, microplastic exposure accelerated female sexual maturity. Transcriptomic sequencing revealed 3464 upregulated and 1100 downregulated genes, with qPCR validation performed on a subset of these genes. The downregulated genes included those associated with the hypothalamus–pituitary–gonadal axis, such as *FSHβ*, *GTHα*, *LHβ*, *Vtg*, and *CHgL*. In contrast, genes involved in the steroid hormone synthesis pathway, including *StAR* and *CYP11a*, were upregulated regardless of the microplastic concentration. These findings collectively demonstrate that microplastic exposure induces significant physiological, molecular, and transcriptomic changes, adversely affecting metabolic processes and reproductive health in aquatic organisms.

## 3. Synergistic Effects of Microplastics and Pollutants on Aquatic Organisms

In addition to microplastics, various pollutants, particularly heavy metals and organic compounds, are prevalent in aquatic environments, each exhibiting inherent toxicity [86,87]. These pollutants readily bind to the highly adsorbent surfaces of microplastics, enabling microplastics to act as vectors for pollutant transportation. While microplastics and pollutants are harmful individually, their combination forms persistent complexes within the marine environment, amplifying ecological damage. These complexes are more likely to be ingested by aquatic organisms, leading to bioaccumulation and severe physiological effects, including neurological, reproductive, and inflammatory responses [32,33,88,89]. This section reviews the impacts of pollutant complexes adsorbed by microplastics on marine life, organized according to the classification criteria established in the previous section.

### 3.1. Phenotypic Alterations in Marine Organisms Due to Microplastic and Pollutant Ingestion

Numerous studies have examined the phenotypes of aquatic organisms exposed to microplastics and other pollutants to understand how co-exposure to these substances impacts aquatic life (Table 5). One study investigated the effects of microplastics and heavy metals on *Hippocampus kuda* [90]. After 14 days, the body length of the group exposed to both stressors decreased compared to the control and, by 35 days, a statistically significant reduction was observed compared to the microplastics-only group. The body weight of the organisms was observed to decrease in the combined exposure group compared to the control after 14 days of exposure and, after 21 days, the decrease was more pronounced in the combined exposure group than in the group exposed to microplastics alone. Antioxidant enzyme activities, including SOD and CAT, as well as the lipid peroxidation marker, MDA, were higher in the combined exposure group than in the microplastics-only group. These findings indicate that combined exposure to heavy metals and microplastics has a more detrimental effect than exposure to microplastics alone. Another study explored the impact of microplastics and cadmium on *Euplotes vannus* [91]. After simultaneous exposure to varying concentrations of microplastics and a constant cadmium concentration, the biomass of the organisms decreased immediately after 60 h in the high-concentration group compared to the cadmium-only control group. When smaller and larger microplastics, compared to those previously used, were combined with cadmium, the biomass of the organisms decreased after 24 h, relative to the control. This effect was more pronounced in the group exposed to smaller microplastics. These results demonstrate that simultaneous exposure to small-sized microplastics and cadmium significantly impacts the biomass of organisms, with higher microplastic concentrations accelerating the effect. Similarly, a study on *Chlorella pyrenoidosa* exposed to microplastics and lead revealed reductions in their chlorophyll a levels and cell growth when subject to individual treatments, with more pronounced reductions occurring when subject to combined exposure [92]. Soluble proteins, crucial for cellular physiological activity, also decreased, following the same trend. Conversely, soluble sugars, a key energy source, and MDA, a cell membrane damage marker, increased significantly when subject to combined exposure, compared to the control. The antioxidant activity also showed a marked increase in the group exposed to both microplastics and lead. These results suggest that combined exposure to microplastics and lead has a synergistic effect, causing more severe adverse impacts on *C*. *pyrenoidosa* than either treatment alone.

### 3.2. Combined Effects of Microplastics and Pollutants on Gene Expression and Function in Aquatic Organisms

Some studies have investigated the effects of microplastics combined with contaminants on aquatic organisms, comparing them to exposure to microplastics or pollutants alone. These studies offer a deeper understanding of how gene expression and function are altered when aquatic organisms are exposed to microplastics adsorbed with other contaminants, providing insight into potential phenotypic changes. Therefore, in this section, we aim to explore the phenotypic, gene expression, and functional effects of co-exposure to microplastics and other pollutants in aquatic organisms, supported by specific examples.

#### 3.2.1. Function of Differentially Expressed Genes Resulting from Microplastic and Pollutant Ingestion in Aquatic Organisms

Few studies have used sequencing to identify the genes whose expression is altered and the functions associated with these changes, although many studies have examined the effects of simultaneous exposure to microplastics and other pollutants on aquatic organisms. The following examples relate to recent findings that examine the phenotypic and molecular effects of simultaneous exposure to microplastics and contaminants, using microarrays and sequencing, to identify genes with altered expression (Table 6). One study examined the phenotypic and genetic changes in *Mytilus galloprovincialis* exposed to microplastics and pyrene [108]. Pyrene accumulation was first assessed in the gills and digestive glands, revealing that pyrene accumulation increased in both tissues when microplastics and pyrene were simultaneously present, compared to microplastic-only treatments. To evaluate the effects of combined exposure to microplastics and pyrene, various biomarkers were analyzed, and the phagocytosis rate and micronuclei/1000 cells significantly increased only in the group exposed to both microplastics and pyrene compared to the microplastics-only group. Microarray analysis was used to compare the genes differentially expressed by microplastics alone with those differentially expressed by the combined exposure to microplastics and pyrene. A total of 1040 genes were found to be differentially expressed only in the combined exposure group, with 544 upregulated and 496 downregulated genes, highlighting the genetic alterations induced by the combined presence of microplastics and pyrene. Another study investigated the effects of microplastics and water-accommodated fractions (WAFs) of crude oil on the growth and reproduction of *Brachionus koreanus* [109]. The exposure to low and high concentrations of microplastics alone showed no significant difference in the growth rate compared to the unexposed control group. However, exposure to WAFs alone reduced the growth rates of the organisms, and this reduction was more pronounced when the WAF was combined with microplastics, with the decrease becoming more severe as the concentration of the microplastics increased. When examining reproduction across multiple generations, no significant differences were observed in the F0, F1, and F2 generations. However, in the F2 generation, a tendency for reduced reproduction was noted in the group exposed to high concentrations of microplastics and WAFs simultaneously. Transcriptome analysis was used to explore the synergistic effects of co-exposure to microplastics and WAFs. The simultaneous exposure group exhibited the differential expression of 4759 genes, with 2581 upregulated and 1980 downregulated genes. Notably, 3791 genes were identified as unique differentially expressed in the co-exposure group. Further investigation into these 3791 genes identified 53 that exhibited synergistic responses to the combination of microplastics and WAFs. Among these, 23 genes were subjected to GO analysis, which revealed that most were associated with metabolism, gene expression, and transportation. This study confirmed that while simultaneous exposure to microplastics and other pollutants did not show a significant difference in terms of the reproduction of the organisms, a decreased tendency was observed in the second generation. This suggests that co-exposure to microplastics and pollutants may have delayed effects that manifest over several generations.

#### 3.2.2. Gene Expression Alterations Due to the Ingestion of Microplastics and Pollutants by Aquatic Organisms

Although sequencing techniques have rarely been employed to identify the effects of simultaneous exposure to microplastics and pollutants, numerous studies have employed qPCR to identify genes with altered expression (Table 7). These studies have also examined the impact of co-exposure by comparing phenotypic and gene expression changes between groups exposed only to microplastics or pollutants and those exposed to both microplastics and pollutants. One study explored the effects of microplastic and copper exposure in *Cyprinus carpio* [33]. In carp exposed to copper alone, the hepatic copper concentrations were elevated, whereas combined exposure to microplastics and copper resulted in even higher hepatic copper levels. Histopathological analysis revealed severe liver lesions in copper-only treated organisms, which were exacerbated when subject to combined exposure to microplastics and copper. Transcriptome analysis revealed that *il1b*, which was overexpressed in the group exposed to copper alone, showed further increased expression in the group co-exposed to microplastics and copper. In contrast, the expression of *cas9*, which remained unchanged between the microplastics-only group and the control, was significantly decreased in the co-treatment group. These findings confirm that co-exposure to microplastics and copper exacerbates the negative effects on inflammatory responses and apoptosis compared to separate single exposures. Another study investigated the effects of microplastics and zinc, both individually and in combination, on *Daphnia magna*, focusing on gender-specific responses [110]. Neonate *Daphnia* exposed to both microplastics and zinc showed significantly reduced survival rates compared to zinc-only treated organisms. In adults, combined exposure resulted in slightly decreased survival rates for both sexes compared to the treatment with zinc alone, with males showing lower survival rates than females. The food ingestion capacity was assessed using *Chlorella vulgaris* as a food source, revealing a decline in the ingestion rates for males subject to co-exposure to microplastics and zinc. For females, the ingestion rates in the combined treatment group were lower than those in the microplastics-only group. The analysis of antioxidant genes (*SOD* and *CAT*) and detoxification-related genes (*GST*) revealed a sex-specific sensitivity: males showed greater sensitivity to *SOD* and *CAT*, whereas females were more responsive to *CAT*. These findings suggest that microplastics may act as mediators for zinc toxicity in aquatic environments, amplifying its adverse effects, with the impact potentially varying between genders. Lastly, a study on *Oryzias melastigma* evaluated the effects of microplastics and phenanthrene co-exposure on phenotypic changes and gene expression [111]. High concentrations of microplastics combined with phenanthrene significantly increased phenanthrene accumulation in the small intestine, uterus, and embryos, compared to phenanthrene-only exposure. Even at low microplastic concentrations, co-exposure led to greater phenanthrene accumulation in embryos than phenanthrene alone. Additionally, high concentrations of microplastics and phenanthrene resulted in increased atretic follicles and reduced vitellogenin levels in the ovary. Nine days post-fertilization, reduced heart rates were observed in all groups exposed to microplastics and phenanthrene, compared to phenanthrene-only treated organisms. Furthermore, the expression of *3βHSD*, *17βHSD*, and *11βHSD* in the ovary varied in the co-exposure groups, confirming that the combination of microplastics and phenanthrene adversely affects the reproductive capacity of organisms.

#### 3.2.3. Modulation of Gene Function and Expression by Microplastics and Pollutants in Aquatic Organisms

Few studies have identified differentially expressed genes through sequencing in aquatic organisms simultaneously exposed to microplastics and pollutants, followed by GO analysis to determine their associated functions (Table 8). Validation with qPCR was subsequently performed to confirm whether the gene expression patterns were consistent with the sequencing results, providing insight into the effects of combined exposure to microplastics and pollutants on marine organisms. For example, one study examined the effects of microplastics and cadmium on *Apostichopus japonicus* by analyzing differentially expressed genes across single and combined treatments [126]. A total of 30 genes were consistently differentially expressed in groups exposed to cadmium alone, cadmium with microplastics, high-concentration cadmium alone, and high-concentration cadmium with microplastics. KEGG enrichment analysis revealed these genes were associated with lipid metabolism, glycan biosynthesis and metabolism, and immune function. Similarly, 27 genes were commonly differentially expressed in groups treated with microplastics alone, microplastics with cadmium, high-concentration microplastics alone, and high-concentration microplastics with cadmium. These genes were linked to endocrine, immune, and digestive systems, as well as lipid metabolism and glycan biosynthesis, with some related to human diseases, such as endocrine and metabolic disorders and infectious diseases. Validation using qPCR showed that the consistent expression patterns were similar. Another study investigated the effects of microplastics and pollutants on *Danio rerio,* by dividing them into groups exposed to copper alone, groups co-exposed to copper and microplastics, and groups exposed to a combination of copper, microplastics, and natural organic matter [127]. The copper accumulation analysis revealed that the copper levels were significantly higher in the group exposed to microplastics combined with copper, compared to the group exposed to copper alone. Additionally, the copper levels were significantly higher in the group exposed to small microplastics, copper, and natural organic matter, than in the other group. A comparison of the genes differentially expressed in the group exposed to copper and microplastics with those in the group exposed to copper, microplastics, and natural organic matter, revealed 81 genes that were commonly differentially expressed. GO enrichment analysis indicated that these genes were primarily associated with metal ion transportation, DNA repair, cell cycle regulation, and the oxidative stress response. Moreover, qPCR validation confirmed the consistent gene expression patterns for those related to metal ion transportation and DNA repair. These findings suggest that microplastics can amplify copper’s biological effects, especially in the presence of natural organic matter, thereby increasing its toxicity.

## 4. Conclusions

**Figure 1:**
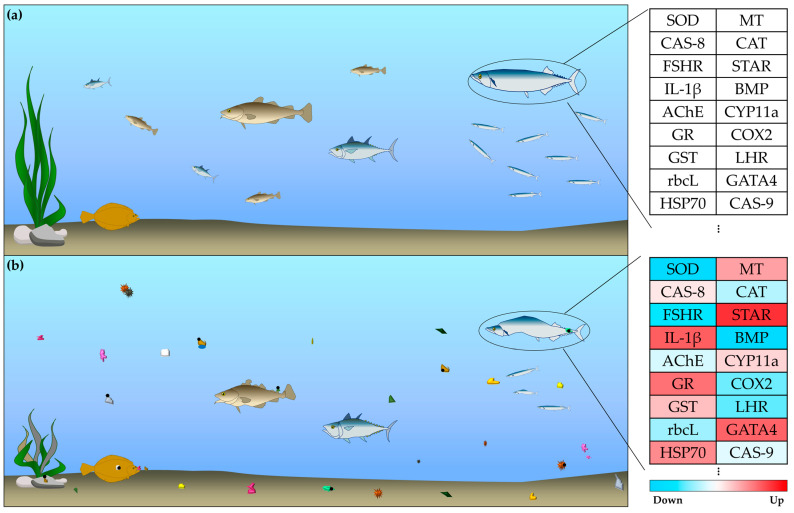
Schematic diagram illustrating the changes in organisms in aquatic environments: (**a**) without microplastics and pollutants and (**b**) with microplastics and pollutants. In an environment with microplastics and pollutants, organisms exhibit phenotypic changes, including malformation, reduced growth rates, and lower hatching success. The table on the right lists some of the genes with altered expression levels, where red indicates upregulation and blue indicates downregulation. Colored shapes: microplastics; black circle: pollutants.

As concern about the hazards of microplastics continues to grow, numerous studies have been actively investigating their effects on aquatic life. Experiments are being conducted under various conditions to better understand how microplastics affect aquatic organisms. These studies have confirmed that microplastics, both alone and in combination with other pollutants, negatively impact the phenotype (e.g., growth, development) and gene expression in aquatic organisms (Figure 1). However, experiments involving multiple variables often yield inconsistent results, making it challenging to confirm the specific effects of microplastics. Furthermore, most experimental studies have focused on short-term exposure, limiting our understanding of the potential long-term impacts of microplastics., given that microplastics, like endocrine disruptors, can persist in the environment for extended periods. Consequently, research is needed to investigate the effects of prolonged or multigenerational exposure to microplastics on aquatic organisms. Research on the gene expression changes induced by microplastics also has some limitations. Sequencing-based studies have primarily identified the general functions of genes with altered expression, while qPCR-based studies have focused on specific genes linked to limited biological functions, such as inflammatory responses. Although some studies have combined sequencing with qPCR techniques, they have largely been confined to confirming whether gene expression is altered, without elucidating the precise pathways through which microplastics exert their effects. To better understand the biological mechanisms affected by microplastics, it is essential to identify the specific biological processes disrupted through sequencing and to verify that the expression patterns of the genes involved in these processes align with sequencing results, using qPCR. This review highlights the limitations of the current research and proposes directions for future studies. Addressing these gaps will enhance our understanding of the mechanisms underlying microplastic toxicity and provide a more comprehensive assessment of the ecological risks posed by these persistent pollutants.

## Figures and Tables

**Table 2:** Information on and findings of experiments assessing the impact of microplastics on aquatic organisms via phenotypic and gene function changes. NA: Not Available.

Microplastic Type	Microplastic Size	Microplastic Concentration	Exposure Time	Phenotype	GO/KEGG Analysis	Experiment Model	Reference
microsphere	1–5 μm	216.67 mg/mL	2 h	NA	adaptation of rhodopsin- mediated signaling, metarhodopsin inactivation, adult somatic muscle development, muscle organ development	*Penaeus vannamei*	[64]
polystyrene	1 μm	50 mg/L	6, 12, 24 h	photosynthetic pigments, antioxidant system, detoxification system, immune system	stress response, zymogen granules, sterol transport, EGF–ERK1/2 signaling pathway	*Pocillopora* *damicornis*	[66]
polyethylene	spherical: 27–32 μm fibrous: 200–400 μm	100 mg/L	4, 14 days	NA	protein modification, transcriptional regulation, metabolic function, signal transduction	*Mytilus* *galloprovincialis*	[31]
polystyrene, polymethyl methacrylate	65, 100 nm, 1 μm	10 mg/L	1, 12, 24, 36, 48, 60, 72 h	cell viability, apoptosis, integrity of the cell membrane	catalytic activity, structural molecular activity, metabolic process, cellular process	*Karenia* *mikimotoi*	[40]
polystyrene	5, 10 μm	25 μg/L	30 days	ingestion rate, oxygen consumption rate, ammonia-N excretion rate, growth, wet flesh weight-specific growth rate	carbohydrate metabolism, citrate cycle	*Ruditapes* *philippinarum*	[67]
polyethylene	10–45 μm	5, 20 mg/L	24, 48, 72, 96, 120 h post-fertilization, 2, 7, 15 days, 1, 3, 4, 7, 12, 19 days post-fertilization	NA	central and peripheral nervous system, neural development, synapse function, translation, ribosomal and spliceosomal function	*Danio rerio*	[68]
polystyrene	0.1, 5 μm	0.5, 1, 10, 50 mg/L	24, 48, 72, 96 h	growth, photosynthetic pigments, antioxidant system	cellular processes, environmental information processing, carbohydrate metabolism, signal transduction	*Euglena gracilis*	[65]

**Table 3:** Experimental parameters and outcomes for evaluating microplastic effects on aquatic organisms based on phenotypic and gene expression changes. NA: Not Available.

Microplastic Type	Microplastic Size	Microplastic Concentration	Exposure Time	Phenotype	Genes	Experiment model	Reference
polystyrene	5 μm	180 μg/L	7, 21, 25, 90, 240 days post-hatching	body weight, body length, antioxidant system	*ghrh*, *gh*, *ghrb*, *ghra*, *igf1*, *igf2b*, *igf1ra*, *igf1rb*, *igf2r*, *igfbp1*, *nrf2*, *keap1a*, *keap1b*, *sod1*, *sod2*, *cat*, *mt2*	*Oryzias* *melastigma*	[71]
polyvinyl chloride	53–106 μm	1 × 10^3^, 1 × 10^6^ particles/L	15, 18, 30, 36, 48, 60, 66, 84, 90, 102, 138, 156, 174, 192, 210, 228, 246, 264, 282, 318 s, 5, 10 min, 1, 2, 3, 12, 24 h, 5, 8, 11 days	hatching time, heart rate, malformation, oxygen flux	*HIF-1α*, *GATA4*, *NKX2.5*	*Oryzias* *melastigma*	[28]
polypropylene, high-density polyethylene	400–1000 μm	NA	5 h, 1, 3, 7, 20, 63, 78 days	aggregation, colonization	*rbcL*, *UGD*, *UGE*, *UGLD*,	*Chlamydomonas reinhardtii*	[72]
polystyrene, high-density polyethylene	<90 μm	100, 1000 μg/L	20 days	NA	*cyp2p8*, *tcra*	*Danio rerio*	[38]
polyvinyl chloride	100–1000 μm	0.1, 0.5 mg/L	96 h	oxidant system, detoxification system	*IGFBP-1*, *gHR*	*Carassius* *auratus*	[73]
high-density polyethylene	red: 10–22 μm, blue: 45–53 μm, green: 90–106 μm, clear: 212–250 μm, yellow: 500–600 μm	2 mg/L, 11, 110, 1100 particles/L	96 h	tail bent downwards or upwards, erratic movement, seizure	*cyp1a*, *vtg1*	*Danio rerio*	[74]
polycaprolactone, polyhydroxy butyrate, polylactic acid	polycaprolactone: 164.90 ± 99.20 μm polyhydroxy butyrate: 0.64 ± 0.3 μm polylactic acid: 335.00 ± 182.01 μm	1, 5, 10 mg/L	1, 48 h post-fertilization	development	*ARF1*, *Mtase*, *HIF1A*, *PARP-1*, *SDH*, *p53*, *ChE*, *CYP-2UL*, *GST*, *GAPDH*, *PKS*, *SULT1*, *ERCC3*, *hsp56*, *hsp60*, *hsp70*, *NF-Kb*, *P38 MAPK*, *Cytb*, *GS*	*Paracentrotus lividus*	[27]
polystyrene	50 nm, 2 μm	0.5, 0.0001 μg/L, 1, 10, 100 mg/L	30 days	antioxidant system	*USP*, *cat*, *tnf*	*Tigriopus* *japonicus*	[75]
polystyrene	10 μm	2, 20, 200 μg/L	10, 30, 60 days	antioxidant system, abnormal proliferation, disintegration of gills, maturation, sex hormones, number of eggs, body length, heart rate	*mGnRH*, *FSHb*, *LHb*, *Cyp19b*, *FSHR*, *LHR*, *Cyp19a*, *Vtg1*, *Vtg*, *ChgL*, *11bHSD*, *CYP11a*, *17bHSD*, *StAR*, *GTHa*, *11bHSD*, *CYP17a1*, *CYP11a2*, *Vtg2*, *ChgH*, *Era*	*Oryzias* *melastigma*	[32]
polystyrene	94–107 nm	10, 100 μg/L	7, 14, 21, 28, 35 days	oxidative stress, lipid peroxidation, antioxidative system, neuron system, biochemical, hepatic histology, inflammation response	*cat*, *sod1*, *gpx1a*, *gstp1*, *hsp70l*, *ptgs2a*, *ache*	*Danio rerio*	[39]
polyvinylchloride	40–150 μm	100, 500 mg/kg	15, 30 days	immune parameter	*prdx5*, *coxIV*, *ucp1*	*Sparus aurata*	[69]
polystyrene	6 μm	1 × 10^2^, 1 × 10^4^, 1 × 10^6^ particles/L	14 days	body length, distance moved	*il-6*, *il-1β*, *tnf-α*, *jak*, *stat-3*, *nf-κb*, *ccl-11*, *heg1*, *muc2*, *muc7-like*, *muc13*, *muc13-like*, *sod*	*Oryzias* *melastigma*	[76]
polymethylmethacrylate	20–1000 μm	19, 85 mg	1, 2, 3, 4, 9, 13, 17 weeks	weight, specific growth rate, feed conversion ratio	*col1a1*, *ighd*, *rpl7*, *c3-3*, *tmem63b*, *ctrl*	*Oncorhynchus mykiss*	[77]
polystyrene	5 μm	0.2, 2, 4, 20, 40, 80, 100, 160, 200, 300, 320, 400, 500, 600, 640, 1280 mg/L	96 h, 1, 2, 4 weeks	survival rate, heart rate, weight, gonadosomatic index, sex hormones, antioxidant system, testicular system, malformation rate, hatching rate, innate immune system	*StAR*, *17βHSD*, *3βHSD*, *Cu-ZnSOD*, *MnSOD*, *CAT*, *GPX*, *LZM*, *PO*	Freshwater prawn	[78]
polyethylene	sphere: 150–180 μm irregular: 6–350 μm	50, 250 mg/L	10, 20, 30, 40 min	total distance traveled, maximum velocity, antioxidant system	*cat*, *sod3*, *cxcr5*, *casp3*, *tp53*	*Cyprinodon* *variegatus*	[79]
polystyrene	1, 5 μm	2, 10, 50 mg/L	1, 5, 10 days	cumulative growth ratio, daily growth ratio, photosynthetic pigments, extracellular polymeric substances, soluble proteins, antioxidant system	*psbA*, *rbcS*, *rbcL*, *chlL*, *ATPF1B*, *ND1*, *AACP*	*Chlorella* *pyrenoidosa*	[70]
polystyrene	50, 10 μm	20 mg/L	24, 48 h	antioxidant system, abnormal proliferation, disintegration of gills, maturation, sex hormones, number of eggs, body length, heart rate	*gr*, *gst*, *cuznsod*, *mnsod*	*Tigriopus* *japonicus*	[80]
polystyrene	0.25, 8 μm	0.05, 0.5, 5 mg/L, 300 μg/L	168 h, 28 days	antioxidant system	*cat*, *sod*, *hsp70*	*Carassius* *auratus*	[81]

**Table 4:** Scales and outcomes of experiments investigating the effects of microplastics on phenotypic traits, gene expression, and functions in aquatic organisms. NA: Not Available.

Microplastic Type	Microplastic Size	Microplastic Concentration	Exposure Time	Phenotype	GO/KEGG Analysis	Genes	Experiment Model	Reference
polystyrene	NA	1, 25, 50, 75, 100 mg/L	24, 48 h, 1, 2, 7, 14 days	morphology, development, body length, apoptosis, oxidative stress	energy derivation, cellular nitrogen compound metabolic process, arrhythmogenic right ventricular cardiomyopathy, viral myocarditis	*EGR1b*, *titin*, *MHC class l antigen*, *Crammer*, *Pyrimidodiazepine* *synthase*, *Dappudraft_310496*, *Dappudraft_308348*	*Artemia* *salina*	[83]
polystyrene	2 μm	2, 20, 200 μg/L	3, 5, 7, 9, 10, 11, 76, 80, 81, 84, 97, 110, 119, 150 days, 5, 6, 7, 8, 9, 10, 11, 12, 13, 14, 15, 16 days post-fertilization	hatching rate, heartbeat, body length, body weight, gonadosomatic index, sexual maturity, antioxidant stress	neuroactive ligand– receptor interaction, steroid hormone biosynthesis	*FSHβ*, *GTHα*, *LHβ*, *Vtg*, *CHgL*, *StAR*, *CYP11a*	*Oryzias* *melastigma*	[84]
high-density polyethylene, polystyrene	NA	100, 1000 μg/L	20 days	intestinal mucosa, gill epithelium, daily activity rhythm, nocturnal activity	sterol biosynthetic process, steroid metabolic process, steroid biosynthesis pathway, terpenoid backbone biosynthesis	*ltb4r*, *iftm1*, *elovl6*, *ch25h*, *cyp51*	*Danio rerio*	[82]
polystyrene	0.05, 0.50, 6.00 μm	0.1, 1 × 10^3^, 1 × 10^6^ particles/mL	3, 4, 5, 6, 7, 8, 9, 19 days post-fertilization	heartbeat, hatching rate	inflammatory mediator regulation of TRP channels, B cell receptor signaling pathway	*HCE*, *LCE*, *BMP4*, *GATA4*, *NKx2.5*	*Oryzias* *melastigma*	[85]

**Table 5:** Conditions and outcomes confirming phenotypic changes in aquatic organisms exposed to both microplastics and pollutants simultaneously. NA: Not Available.

Microplastic Type	Microplastic Size	Microplastic Concentration	Exposure Time	Pollutants	Pollutant Concentration	Phenotype	Experiment Model	Reference
polyvinyl chloride	NA	NA	14 days	cadmium	0, 5, 15, 25 mg	fresh weight	*Vallisneria natans*	[93]
polyethylene	NA	100 mg/L	15 days	lead	1 mg/L	antioxidant system, lipid peroxidation, inflammatory signaling	*Clarias gariepinus*	[94]
polytetrafluoroethylene	2–20 μm	20 mg/L	0.5, 1, 3, 7, 12, 24, 36, 48, 60, 72, 96 h	copper	0.5, 1, 2, 3, 5, 8, 10 mg/L	growth, photosynthetic pigments, antioxidant system	*Microcystis* *aeruginosa*	[95]
high-density polyethylene	NA	NA	0.5, 1, 1.5, 2, 4, 6, 8, 10, 12 h, 7, 14, 15, 21, 28, 30, 35, 42, 45 days	copper, cadmium, lead	copper: 0.05 mg/L cadmium: 0.01 mg/L lead: 0.05 mg/L	body length, condition factor, body weight, antioxidant system, lipid peroxidation	*Hippocampus kuda*	[90]
polyethylene	250–300 μm	1 mg/mL	72 h, 3, 7, 14, 21, 28 days	silver, copper, chromium	silver: 0.001 mg/L, copper: 0.05 mg/L, chromium: 0.5 mg/L	growth, photosynthesis activity, oxidative stress	*Scenedesmus armatus*, *Microcystis* *aeruginosa*	[96]
polyethylene	250–300 μm	NA	3, 7, 14, 21, 28 days	amoxicillin, ibuprofen, sertraline, simazine	NA	growth, photosynthetic activity	*Scenedesmus armatus*, *Microcystis* *aeruginosa*	[97]
polystyrene	NA	1 mg/L	2, 4, 6, 8 days	lead	0.05, 0.1, 0.2, 0.5 mg/L	growth, photosynthetic pigments, antioxidant system, ultrastructure	*Microcystis* *aeruginosa*	[98]
polyamide	5–50 μm	25–250 mg/L	NA	bisphenol a	5, 7.5, 10, 12.5, 15, 20 mg/L	immobilization	*Daphnia magna*	[99]
polyethylene	4–6 μm	10 mg/L	NA	benzo(a)pyrene, perfluorooctanesulfonic acid, benzophenone-3	benzo(a)pyrene: 0.01 and 16.64 μg/g perfluorooctanesulfonic acid: 0.12, 55.65 µg/g benzophenone-3: 0.14, 24 ng/g	embryonic survival, hatching success, larvae head length, total length, abnormal individuals, distance swam, velocity	*Oryzias* *melastigma*	[100]
polyethylene	10–90 µm	0–25,000 MP/mL	48 h	triclosan	300 µg/L	mortality	*Acartia tonsa*	[101]
polyethylene	15–25 μm	500, 1000 μg/L	15 days	lead	2.5, 5 mg/L	hepatotoxicity, neurotoxicity, antioxidant system, metabolism	*Caridina* *fossarum*	[102]
polystyrene	0.1 μm	10, 100 µg/L	1, 2, 3, 4, 6, 8, 10, 12, 14 days	roxithromycin	50 µg/L	neurotoxicity, antioxidant system, cytochrome activity	*Oreochromis niloticus*	[103]
polyethylene, polystyrene, polyvinyl chloride	polyethylene, polystyrene, polyvinyl chloride: 74 μm polyvinyl chloride 800: 1 μm	0.01, 0.02, 0.05, 0.1 g/L	24, 48, 72, 96 h	triclosan	0.1, 0.2, 0.3, 0.4 mg/L	growth, antioxidant system, lipid peroxidation	*Skeletonema costatum*	[104]
polystyrene	1.07, 2.14, 5 μm	2 × 10^5^, 2 × 10^6^, 4 × 10^6^, 6 × 10^6^ items/mL	12, 24, 36, 48, 60, 72, 84, 96 h	cadmium	22.5, 45, 57.6, 67.5, 90 mg/L	antioxidant system, lipid peroxidation	*Euplotes* *vannus*	[91]
polyethylene terephthalate	aged: 20–50 nm; virgin: 100, 300 nm	0.8 mg/L	1, 2, 4, 8, 14, 24, 36 h	lead	2 µg/mL	growth, photosynthetic pigments, antioxidant system, lipid peroxidation, soluble proteins, soluble sugars	*Chlorella* *pyrenoidosa*	[92]
polystyrene, polyvinyl chloride	150, 250 μm	0.01, 0.1, 1 g/L	1, 2, 3, 4, 5, 6, 7 days	copper, cadmium	0.5, 1, 2 mg/L	antioxidant system, lipid peroxidation	*Chlorella* *vulgaris*	[105]
polystyrene	100 nm, 5 μm	10, 20, 50, 100 mg/L	0.5, 1, 2, 4, 5, 6, 8, 15, 24, 30, 48, 60, 72, 120, 150, 180 h	arsenic	10, 20, 30, 40, 50, 75, 100, 150 mg/L	growth, photosynthesis, respiration	*Chlamydomonas* *reinhardtii*	[106]
polypropylene, polystyrene, polyvinyl chloride	<100 μm	0.1, 0.2, 0.4, 1 g/L	24, 36, 48, 72, 96 h	lead, copper, chromium, cadmium	50, 500, 1000 μg/L	cell density, antioxidant system, growth	*Chlorella* *vulgaris*	[107]

**Table 6:** Experimental frameworks and findings from analyzing phenotypic and gene function in aquatic organisms exposed to microplastics and pollutants. NA: Not Available.

Microplastic Type	Microplastic Size	Microplastic Concentration	Exposure Time	Pollutants	Pollutant Concentration	Phenotype	GO/KEGG Analysis	Experiment Model	Reference
polyethylene, polystyrene	<100 μm	20 g/L	3, 6 days	pyrene	0.5, 5, 50 µg/L	phagocytosis rate, micronuclei/1000 cells	NA	*Mytilus* *galloprovincialis*	[108]
polystyrene	0.05 μm	0.1, 1 µg/mL	1, 2, 3, 4, 5, 6, 7, 8 days	water-accommodated fractions	NA	growth rate	mRNA processing, peptide biosynthesis	*Brachionus* *koreanus*	[109]

**Table 7:** Experimental design and results on verifying the phenotypic and gene expression alterations in aquatic organisms exposed to both microplastics and pollutants. NA: Not Available.

Microplastic Type	Microplastic Size	Microplastic Concentration	Exposure Time	Pollutants	Pollutant Concentration	Phenotype	Genes	Experiment Model	Reference
polyvinyl chloride	140.7 ± 5.11 μm	0.5 mg/L	14 days	copper	0.25 mg/L	hepatic histopathology, number of melanomacrophage centers, melanomacrophage center area	*hsp70*, *tnfa*, *il1b*, *cyp1a1*, *gst*, *cas3*, *cas9*	*Cyprinus* *carpio*	[33]
polystyrene	2.5 μm	100 μg/L	NA	cadmium, lead, zinc	100 mg/L	gut microbiota, gonadal development	*mGnRH*, *GnRHR*, *B-AR-α*, *L-AR-α*, *L-ER-α*, *L-ER-β*, *VTG1*, *ChgL*, *G-ER-α*	*Oryzias* *melastigma*	[112]
microspheres	1–5 mm	2 mg/L	2, 6, 10, 14 days post-fertilization	copper	60, 125 mg/L	mortality, oxidative stress, antioxidant system	*cat*, *gstp1*, *mt2*, *ache*	*Danio rerio*	[113]
polystyrene	5 μm	500 μg/L	6, 12, 18, 24 h, 30 days	cadmium	5 μg/L	body weight, antioxidant system	*keap1b*, *igf1rb*, *igfbp5a*, *nrf2*, *mt2*, *hsp70*, *igfbp1a*, *bcl2*, *ghra*, *igf1*, *igf1ra*, *igfbp2b*, *igfbp6a*	*Danio rerio*	[114]
polystyrene	2, 6 μm	32 mg/L	7, 14 days	fluoranthene	30 mg/L	histopathological lesions/ abnormalities, oxidative stress, antioxidant system	*cat*, *pk*, *sod*	*Mytilus* *edulis*, *Mytilus galloprovincialis*	[115]
polyethylene, polyethylene terephthalate, polypropylene, polyethylene vinyl acetate, high-density polyethylene,	<100 µm	50 µg/L	1, 3 days	benzo[a]pyrene	1 µg/L	micronuclei frequency, DNA fragmentation	*DNA ligase*, *bax*, *cas-3*, *p53*,	*Mytilus* *galloprovincialis*	[116]
low-density polyethylene	20–25 µm	10 mg/L	7, 14, 28 days	benzo[a]pyrene	15 µg/g, 150 ng/L	immune, DNA strand breaks	*hsp70*	*Mytilus* *galloprovincialis*	[117]
polystyrene	2 μm	1, 10 mg/L	48, 96 h, 21 days	zinc	0.5, 0.75, 1, 1.5, 2, 3, 4, 5, 6, 10 mg/L	survival rate, chlorella ingestion, total fecundity, days of the first brood, antennae beating, oxidative stress, antioxidant system	*SOD*, *CAT*, *GST*, *ABC transporter*	*Daphnia magna*	[110]
polystyrene	10 μm	0, 1, 10, 20, 50, 100, 200 mg/L	1, 2, 3, 4, 5, 6, 7, 8 min, 2, 3, 4, 5, 6, 7 days post-amputation	lead	0.1, 0.2, 0.3, 0.4, 0.5, 0.6, 1 mg/L	regeneration, antioxidant system, DNA integrity, energy metabolism, ferroptosis	*Cu-Zn SOD*, *GST*, *GPX*, *nak*, *p53*, *cas-3*	*Dugesia* *japonica*	[118]
microsphere	1–5 µm	0.3 mg/L	3, 9 days post-fertilization	copper	10, 30, 90, 270, 810 µg/L	NA	*cat*, *ache*	*Pagellus* *bogaraveo*	[88]
polystyrene	10 μm	2, 20, 200 μg/L	2 days post-hatching, 1, 2, 3, 4, 5, 6, 7, 8, 9, 10, 11, 12, 13 days post-fertilization	phenanthrene	50 μg/L	deformity rate, hatching time, hatching rate, body length, body weight, death rate, heartbeat	*GATA*, *BMP*, *COX*, *SmyD1*, *EPO*, *NKX2.5*	*Oryzias* *melastigma*	[119]
polystyrene	5 μm	20, 200 mg/L	3 weeks	cadmium	100 mg/L	antioxidant system, metal detoxification	*nfe212*, *mt1*, *mt2*, *tnfa*, *il1b*, *ifng1-2*	*Danio rerio*	[120]
virgin polystyrene	80 nm	50, 500 μg/L	24, 48, 96 h	cadmium	50 μg/L	bending of gill lamellae	*IL-1β*, *TNF-α*, *MT*, *HSP70*	*Channa* *maculata*, *Channa* *argus*	[121]
polystyrene	6 μm	2.5, 5, 10, 20, 30 mg/L	24, 48 h	chromium	0.1, 0.2, 0.3, 0.4, 0.5, 0.6, 0.7, 0.8, 0.9, 1.0 mg/L	adult survival rate, first neonate body length, antioxidant system	*PGC-1α*, *Drp1*, *ABCB1*, *ABCB7*, *ABCC4-1*, *ABCC9-1*	*Daphnia magna*	[122]
carboxyl -modified polystyrene	2 μm	1, 10 μg/mL	48 h	triclosan	100, 150, 200, 250, 300, 330, 360, 400, 425, 450, 500 μg/L	survival rate, total fecundity, first brood day, heart rate, oxidative stress, antioxidant system	*ABCC4-3*	*Daphnia magna*	[123]
polystyrene	NA	400 μg/L	7, 14, 21 days	lead	5, 50 μg/L	antioxidant system, lipid metabolism, histopathological changes	*ACC*, *Elovl6*, *FAD6b*	*Eriocheir* *sinensis*	[124]
polystyrene	NA	2, 20, 200 μg/L	60 days	phenanthrene	50 µg/L	histopathological changes, atretic follicles, heartbeat, body width	*3βHSD*, *17βHSD*, *11βHSD*	*Oryzias* *melastigma*	[111]
polystyrene	80 nm, 0.5 μm	200 μg/L	24, 48, 96 h	cadmium	50 μg/L	antioxidant system	*IL-1β*, *HSP70*, *MT*	*Channa* *argus*	[125]

**Table 8:** Outline of experimental conditions and results from evaluating the effects of co-exposure to microplastics and pollutants on phenotype, gene expression, and function in aquatic organisms. NA: Not Available.

Microplastic Type	Microplastic Size	Microplastic Concentration	Exposure Time	Pollutants	Pollutant Concentration	Phenotype	GO/KEGG Analysis	Genes	Experiment Model	Reference
polyethylene glycol terephthalate	NA	1000, 100,000 particles/kg	30 days	cadmium	0.5, 50 mg/kg	antioxidant system	lipid metabolism, immune system, glycan biosynthesis, glycan metabolism	*BSL78_01257*, *BSL78_04100*, *BSL78_07802*, *BSL78_08543*, *BSL78_12019*, *BSL78_20141*	*Apostichopus japonicus*	[126]
polystyrene	0.1, 20 μm	40 mg/L	3, 6, 12 h, 1, 2, 4, 6, 8, 10, 14 days	copper, natural organic matter	copper: 5 mg/L; natural organic matter: 5 mg/L	antioxidant system	metal ion transport, DNA repair, cell cycle regulation, oxidative stress response	*LOXA*, *COX4I1*, *MAT2AB*, *ABCA12*, *ABCB5*, *KIF20B*, *RAD52*, *LMX1BA*, *MIOX. DHRS7CB*	*Danio rerio*	[127]

## References

[1] Bishop G., Styles D., Lens P.N. (2020). Recycling of European plastic is a pathway for plastic debris in the ocean. Environ. Int..

[2] Zhao K., Wei Y., Dong J., Zhao P., Wang Y., Pan X., Wang J. (2022). Separation and characterization of microplastic and nanoplastic particles in marine environment. Environ. Pollut..

[3] Su L., Xiong X., Zhang Y., Wu C., Xu X., Sun C., Shi H. (2022). Global transportation of plastics and microplastics: A critical review of pathways and influences. Sci. Total Environ..

[4] Ahmad J., Majdi A., Babeker Elhag A., Deifalla A.F., Soomro M., Isleem H.F., Qaidi S. (2022). A step towards sustainable concrete with substitution of plastic waste in concrete: Overview on mechanical, durability and microstructure analysis. Crystals.

[5] Our World in Data. https://ourworldindata.org/plastic-pollution.

[6] Hurley R., Horton A., Lusher A., Nizzetto L. (2020). Plastic waste in the terrestrial environment. Plastic Waste and Eecycling.

[7] Wayman C., Niemann H. (2021). The fate of plastic in the ocean environment—A minireview. Environ. Sci. Process. Impacts.

[8] Li J., Liu H., Chen J.P. (2018). Microplastics in freshwater systems: A review on occurrence, environmental effects, and methods for microplastics detection. Water Res..

[9] Liu F., Vianello A., Vollertsen J. (2019). Retention of microplastics in sediments of urban and highway stormwater retention ponds. Environ. Pollut..

[10] Martin J., Lusher A., Thompson R.C., Morley A. (2017). The deposition and accumulation of microplastics in marine sediments and bottom water from the Irish continental shelf. Sci. Rep..

[11] Cole M., Lindeque P., Halsband C., Galloway T.S. (2011). Microplastics as contaminants in the marine environment: A review. Mar. Pollut. Bull..

[12] Tang K.H.D., Hadibarata T. (2021). Microplastics removal through water treatment plants: Its feasibility, efficiency, future prospects and enhancement by proper waste management. Environ. Chall..

[13] Plastic Waste Accumulated in Aquatic Environments, World. https://ourworldindata.org/grapher/plastic-leakage-to-aquatic-environments?country=~OWID_WRL.

[14] Berenstein G., Córdoba P., Díaz Y.B., González N., Ponce M.B., Montserrat J.M. (2024). Macro, meso, micro and nanoplastics in horticultural soils in Argentina: Abundance, size distribution and fragmentation mechanism. Sci. Total Environ..

[15] Gola D., Tyagi P.K., Arya A., Chauhan N., Agarwal M., Singh S., Gola S. (2021). The impact of microplastics on marine environment: A review. Environ. Nanotechnol. Monit. Manag..

[16] Wagner M., Scherer C., Alvarez-Muñoz D., Brennholt N., Bourrain X., Buchinger S., Fries E., Grosbois C., Klasmeier J., Marti T. (2014). Microplastics in freshwater ecosystems: What we know and what we need to know. Environ. Sci. Eur..

[17] Loganathan Y., Kizhakedathil M.P.J. (2023). A review on microplastics-an indelible ubiquitous pollutant. Biointerface Res. Appl. Chem..

[18] Ziani K., Ioniță-Mîndrican C.-B., Mititelu M., Neacșu S.M., Negrei C., Moroșan E., Drăgănescu D., Preda O.-T. (2023). Microplastics: A real global threat for environment and food safety: A state of the art review. Nutrients.

[19] Zhou C., Bi R., Su C., Liu W., Wang T. (2022). The emerging issue of microplastics in marine environment: A bibliometric analysis from 2004 to 2020. Mar. Pollut. Bull..

[20] Laskar N., Kumar U. (2019). Plastics and microplastics: A threat to environment. Environ. Technol. Innov..

[21] Hidayaturrahman H., Lee T.-G. (2019). A study on characteristics of microplastic in wastewater of South Korea: Identification, quantification, and fate of microplastics during treatment process. Mar. Pollut. Bull..

[22] Song J., Wang C., Li G. (2024). Defining Primary and Secondary Microplastics: A Connotation Analysis. ACS ES&T Water.

[23] Periyasamy A.P., Tehrani-Bagha A. (2022). A review on microplastic emission from textile materials and its reduction techniques. Polym. Degrad. Stab..

[24] Hossain M.B., Pingki F.H., Azad M.A.S., Nur A.A.U., Banik P., Paray B.A., Arai T., Yu J. (2023). Microplastics in Different Tissues of a Commonly Consumed Fish, *Scomberomorus guttatus*, from a Large Subtropical Estuary: Accumulation, Characterization, and Contamination Assessment. Biology.

[25] Huang J.S., Koongolla J.B., Li H.X., Lin L., Pan Y.F., Liu S., He W., Maharana D., Xu X.R. (2020). Microplastic accumulation in fish from Zhanjiang mangrove wetland, South China. Sci. Total Environ..

[26] Aldana Arana D., Gil Cortés T.P., Castillo Escalante V., Rodríguez-Martínez R.E. (2024). Pelagic Sargassum as a Potential Vector for Microplastics into Coastal Ecosystems. Phycology.

[27] Viel T., Cocca M., Manfra L., Caramiello D., Libralato G., Zupo V., Costantini M. (2023). Effects of biodegradable-based microplastics in Paracentrotus lividus Lmk embryos: Morphological and gene expression analysis. Environ. Pollut..

[28] Xia B., Sui Q., Du Y., Wang L., Jing J., Zhu L., Zhao X., Sun X., Booth A.M., Chen B. (2022). Secondary PVC microplastics are more toxic than primary PVC microplastics to *Oryzias melastigma* embryos. J. Hazard. Mater..

[29] Naidoo T., Glassom D. (2019). Decreased growth and survival in small juvenile fish, after chronic exposure to environmentally relevant concentrations of microplastic. Mar. Pollut. Bull..

[30] Nugnes R., Russo C., Lavorgna M., Orlo E., Kundi M., Isidori M. (2022). Polystyrene microplastic particles in combination with pesticides and antiviral drugs: Toxicity and genotoxicity in *Ceriodaphnia dubia*. Environ. Pollut..

[31] Rangaswamy B., An J., Kwak I.-S. (2024). Different recovery patterns of the surviving bivalve *Mytilus galloprovincialis* based on transcriptome profiling exposed to spherical or fibrous polyethylene microplastics. Heliyon.

[32] Wang J., Li Y., Lu L., Zheng M., Zhang X., Tian H., Wang W., Ru S. (2019). Polystyrene microplastics cause tissue damages, sex-specific reproductive disruption and transgenerational effects in marine medaka (*Oryzias melastigma*). Environ. Pollut..

[33] Hoseini S.M., Khosraviani K., Delavar F.H., Arghideh M., Zavvar F., Hoseinifar S.H., Van Doan H., Zabihi E., Reverter M. (2022). Hepatic transcriptomic and histopathological responses of common carp, *Cyprinus carpio*, to copper and microplastic exposure. Mar. Pollut. Bull..

[34] Guzzetti E., Sureda A., Tejada S., Faggio C. (2018). Microplastic in marine organism: Environmental and toxicological effects. Environ. Toxicol. Pharmacol..

[35] Collard F., Gilbert B., Compère P., Eppe G., Das K., Jauniaux T., Parmentier E. (2017). Microplastics in livers of European anchovies (*Engraulis encrasicolus*, L.). Environ. Pollut..

[36] Mallik A., Xavier K.M., Naidu B.C., Nayak B.B. (2021). Ecotoxicological and physiological risks of microplastics on fish and their possible mitigation measures. Sci. Total Environ..

[37] Li J., Liu X., Fu J., Gong Z., Jiang S.Y., Chen J.P. (2024). Metabolic profile changes of zebrafish larvae in the single-and co-exposures of microplastics and phenanthrene. Sci. Total Environ..

[38] Limonta G., Mancia A., Abelli L., Fossi M.C., Caliani I., Panti C. (2021). Effects of microplastics on head kidney gene expression and enzymatic biomarkers in adult zebrafish. Comp. Biochem. Physiol. Part C Toxicol. Pharmacol..

[39] Umamaheswari S., Priyadarshinee S., Bhattacharjee M., Kadirvelu K., Ramesh M. (2021). Exposure to polystyrene microplastics induced gene modulated biological responses in zebrafish (*Danio rerio*). Chemosphere.

[40] Zhao T., Tan L., Han X., Wang X., Zhang Y., Ma X., Lin K., Wang R., Ni Z., Wang J. (2022). Microplastic-induced apoptosis and metabolism responses in marine Dinoflagellate, *Karenia mikimotoi*. Sci. Total Environ..

[41] Yin L., Chen B., Xia B., Shi X., Qu K. (2018). Polystyrene microplastics alter the behavior, energy reserve and nutritional composition of marine jacopever (*Sebastes schlegelii*). J. Hazard. Mater..

[42] Jabeen K., Li B., Chen Q., Su L., Wu C., Hollert H., Shi H. (2018). Effects of virgin microplastics on goldfish (*Carassius auratus*). Chemosphere.

[43] Trifuoggi M., Pagano G., Oral R., Pavičić-Hamer D., Burić P., Kovačić I., Siciliano A., Toscanesi M., Thomas P.J., Paduano L. (2019). Microplastic-induced damage in early embryonal development of sea urchin *Sphaerechinus granularis*. Environ. Res..

[44] Muhr J., Ackerman K.M. (2023). Embryology, Gastrulation.

[45] Ziajahromi S., Kumar A., Neale P.A., Leusch F.D. (2017). Impact of microplastic beads and fibers on waterflea (*Ceriodaphnia dubia*) survival, growth, and reproduction: Implications of single and mixture exposures. Environ. Sci. Technol..

[46] Nasser A.M., Sheekh M.M.E., Zeineldein M.H., Al Maghraby D.M., Hassan I.A. (2022). Physiological, morphological, and growth effects of microplastics on freshwater alga *Chlorella vulgaris*. Rend. Lincei Sci. Fis. Nat..

[47] Nugnes R., Lavorgna M., Orlo E., Russo C., Isidori M. (2022). Toxic impact of polystyrene microplastic particles in freshwater organisms. Chemosphere.

[48] Menezes M., de Mello F.T., Ziegler L., Wanderley B., Gutiérrez J.M., Dias J.D. (2024). Revealing the hidden threats: Genotoxic effects of microplastics on freshwater fish. Aquat. Toxicol..

[49] Senavirathna M.D.H.J., Zhaozhi L., Fujino T. (2022). Root adsorption of microplastic particles affects the submerged freshwater macrophyte *Egeria densa*. Water Air Soil Pollut..

[50] Jeyavani J., Sibiya A., Gopi N., Mahboob S., Riaz M.N., Vaseeharan B. (2022). Dietary consumption of polypropylene microplastics alter the biochemical parameters and histological response in freshwater benthic mollusc *Pomacea paludosa*. Environ. Res..

[51] Jeyavani J., Sibiya A., Stalin T., Vigneshkumar G., Al-Ghanim K.A., Riaz M.N., Govindarajan M., Vaseeharan B. (2023). Biochemical, genotoxic and histological implications of polypropylene microplastics on freshwater fish Oreochromis mossambicus: An aquatic eco-toxicological assessment. Toxics.

[52] Au S.Y., Bruce T.F., Bridges W.C., Klaine S.J. (2015). Responses of *Hyalella azteca* to acute and chronic microplastic exposures. Environ. Toxicol. Chem..

[53] Murphy F., Quinn B. (2018). The effects of microplastic on freshwater *Hydra attenuata* feeding, morphology & reproduction. Environ. Pollut..

[54] Wu Y., Guo P., Zhang X., Zhang Y., Xie S., Deng J. (2019). Effect of microplastics exposure on the photosynthesis system of freshwater algae. J. Hazard. Mater..

[55] Li S., Wang P., Zhang C., Zhou X., Yin Z., Hu T., Hu D., Liu C., Zhu L. (2020). Influence of polystyrene microplastics on the growth, photosynthetic efficiency and aggregation of freshwater microalgae *Chlamydomonas reinhardtii*. Sci. Total Environ..

[56] Xu H., Wang Y., Qiu K., Chen S., Zeng J., Liu R., Yang Q., Huang W. (2023). Differential physiological response of marine and freshwater microalgae to polystyrene microplastics. J. Hazard. Mater..

[57] Natarajan L., Soupam D., Dey S., Chandrasekaran N., Kundu R., Paul S., Mukherjee A. (2022). Toxicity of polystyrene microplastics in freshwater algae *Scenedesmus obliquus*: Effects of particle size and surface charge. Toxicol. Rep..

[58] Harrison P.W., Wright A.E., Mank J.E. (2012). The evolution of gene expression and the transcriptome–phenotype relationship. Seminars in Cell & Developmental Biology.

[59] Hanczar B., Zehraoui F., Issa T., Arles M. (2020). Biological interpretation of deep neural network for phenotype prediction based on gene expression. BMC Bioinform..

[60] Prinz N., Korez Š. (2020). Understanding how microplastics affect marine biota on the cellular level is important for assessing ecosystem function: A review. YOUMARES 9-The Oceans: Our Research, Our Future, Proceedings of the 2018 Conference for Young Marine Researcher in Oldenburg, Germany, 11–14 September 2020.

[61] Freitas F.C., Depintor T.S., Agostini L.T., Luna-Lucena D., Nunes F.M., Bitondi M.M., Simões Z.L., Lourenço A.P. (2019). Evaluation of reference genes for gene expression analysis by real-time quantitative PCR (qPCR) in three stingless bee species (Hymenoptera: Apidae: Meliponini). Sci. Rep..

[62] Harshitha R., Arunraj D.R. (2021). Real-time quantitative PCR: A tool for absolute and relative quantification. Biochem. Mol. Biol. Educ..

[63] Finotello F., Di Camillo B. (2015). Measuring differential gene expression with RNA-seq: Challenges and strategies for data analysis. Brief. Funct. Genomics.

[64] Han J.E., Choi S.-K., Jeon H.J., Park J.-K., Han S.-H., Jeong J., Kim J.H., Lee J. (2021). Transcriptional response in the whiteleg shrimp (*Penaeus vannamei*) to short-term microplastic exposure. Aquac. Rep..

[65] Xiao Y., Jiang X., Liao Y., Zhao W., Zhao P., Li M. (2020). Adverse physiological and molecular level effects of polystyrene microplastics on freshwater microalgae. Chemosphere.

[66] Tang J., Ni X., Zhou Z., Wang L., Lin S. (2018). Acute microplastic exposure raises stress response and suppresses detoxification and immune capacities in the scleractinian coral *Pocillopora damicornis*. Environ. Pollut..

[67] Jiang W., Fang J., Du M., Gao Y., Fang J., Jiang Z. (2022). Microplastics influence physiological processes, growth and reproduction in the Manila clam, *Ruditapes philippinarum*. Environ. Pollut..

[68] LeMoine C.M., Kelleher B.M., Lagarde R., Northam C., Elebute O.O., Cassone B.J. (2018). Transcriptional effects of polyethylene microplastics ingestion in developing zebrafish (*Danio rerio*). Environ. Pollut..

[69] Espinosa C., Cuesta A., Esteban M.Á. (2017). Effects of dietary polyvinylchloride microparticles on general health, immune status and expression of several genes related to stress in gilthead seabream (*Sparus aurata* L.). Fish Shellfish Immunol..

[70] Cao Q., Sun W., Yang T., Zhu Z., Jiang Y., Hu W., Wei W., Zhang Y., Yang H. (2022). The toxic effects of polystyrene microplastics on freshwater algae *Chlorella pyrenoidosa* depends on the different size of polystyrene microplastics. Chemosphere.

[71] Zhang X., Chen X., Gao L., Zhang H.-T., Li J., Ye Y., Zhu Q.-L., Zheng J.-L., Yan X. (2024). Transgenerational effects of microplastics on Nrf2 signaling, GH/IGF, and HPI axis in marine medaka *Oryzias melastigma* under different salinities. Sci. Total Environ..

[72] Lagarde F., Olivier O., Zanella M., Daniel P., Hiard S., Caruso A. (2016). Microplastic interactions with freshwater microalgae: Hetero-aggregation and changes in plastic density appear strongly dependent on polymer type. Environ. Pollut..

[73] Romano N., Renukdas N., Fischer H., Shrivastava J., Baruah K., Egnew N., Sinha A.K. (2020). Differential modulation of oxidative stress, antioxidant defense, histomorphology, ion-regulation and growth marker gene expression in goldfish (*Carassius auratus*) following exposure to different dose of virgin microplastics. Comp. Biochem. Physiol. C Toxicol. Pharmacol..

[74] Mak C.W., Yeung K.C.-F., Chan K.M. (2019). Acute toxic effects of polyethylene microplastic on adult zebrafish. Ecotoxicol. Environ. Saf..

[75] Kim K., Yoon H., Choi J.S., Jung Y.-J., Park J.-W. (2022). Chronic effects of nano and microplastics on reproduction and development of marine copepod *Tigriopus japonicus*. Ecotoxicol. Environ. Saf..

[76] Chen J.-C., Fang C., Zheng R.-H., Chen M.-L., Kim D.-H., Lee Y.-H., Bailey C., Wang K.-J., Lee J.-S., Bo J. (2022). Environmentally relevant concentrations of microplastics modulated the immune response and swimming activity, and impaired the development of marine medaka *Oryzias melastigma* larvae. Ecotoxicol. Environ. Saf..

[77] Roch S., Rebl A., Wolski W., Brinker A. (2022). Combined proteomic and gene expression analysis to investigate reduced performance in rainbow trout (*Oncorhynchus mykiss*) caused by environmentally relevant microplastic exposure. Microplast. nanoplast..

[78] Sun S., Jin Y., Luo P., Shi X. (2022). Polystyrene microplastics induced male reproductive toxicity and transgenerational effects in freshwater prawn. Sci. Total Environ..

[79] Choi J.S., Jung Y.-J., Hong N.-H., Hong S.H., Park J.-W. (2018). Toxicological effects of irregularly shaped and spherical microplastics in a marine teleost, the sheepshead minnow (*Cyprinodon variegatus*). Mar. Pollut. Bull..

[80] Choi J.S., Hong S.H., Park J.-W. (2020). Evaluation of microplastic toxicity in accordance with different sizes and exposure times in the marine copepod *Tigriopus japonicus*. Mar. Environ. Res..

[81] Abarghouei S., Hedayati A., Raeisi M., Hadavand B.S., Rezaei H., Abed-Elmdoust A. (2021). Size-dependent effects of microplastic on uptake, immune system, related gene expression and histopathology of goldfish (*Carassius auratus*). Chemosphere.

[82] Limonta G., Mancia A., Benkhalqui A., Bertolucci C., Abelli L., Fossi M.C., Panti C. (2019). Microplastics induce transcriptional changes, immune response and behavioral alterations in adult zebrafish. Sci. Rep..

[83] Suman T.Y., Jia P.-P., Li W.-G., Junaid M., Xin G.-Y., Wang Y., Pei D.-S. (2020). Acute and chronic effects of polystyrene microplastics on brine shrimp: First evidence highlighting the molecular mechanism through transcriptome analysis. J. Hazard. Mater..

[84] Wang J., Zheng M., Lu L., Li X., Zhang Z., Ru S. (2021). Adaptation of life-history traits and trade-offs in marine medaka (*Oryzias melastigma*) after whole life-cycle exposure to polystyrene microplastics. J. Hazard. Mater..

[85] Chen J.-C., Chen M.-Y., Fang C., Zheng R.-H., Jiang Y.-L., Zhang Y.-S., Wang K.-J., Bailey C., Segner H., Bo J. (2020). Microplastics negatively impact embryogenesis and modulate the immune response of the marine medaka *Oryzias melastigma*. Mar. Pollut. Bull..

[86] Vieira K.S., Neto J.A.B., Crapez M.A.C., Gaylarde C., da Silva Pierri B., Saldaña-Serrano M., Bainy A.C.D., Nogueira D.J., Fonseca E.M. (2021). Occurrence of microplastics and heavy metals accumulation in native oysters Crassostrea Gasar in the Paranaguá estuarine system, Brazil. Mar. Pollut. Bull..

[87] Mei W., Chen G., Bao J., Song M., Li Y., Luo C. (2020). Interactions between microplastics and organic compounds in aquatic environments: A mini review. Sci. Total Environ..

[88] Santos D., Perez M., Perez E., Cabecinha E., Luzio A., Félix L., Monteiro S.M., Bellas J. (2022). Toxicity of microplastics and copper, alone or combined, in blackspot seabream (*Pagellus bogaraveo*) larvae. Environ. Toxicol. Pharmacol..

[89] Tang Y., Rong J., Guan X., Zha S., Shi W., Han Y., Du X., Wu F., Huang W., Liu G. (2020). Immunotoxicity of microplastics and two persistent organic pollutants alone or in combination to a bivalve species. Environ. Pollut..

[90] Jinhui S., Sudong X., Yan N., Xia P., Jiahao Q., Yongjian X. (2019). Effects of microplastics and attached heavy metals on growth, immunity, and heavy metal accumulation in the yellow seahorse, *Hippocampus kuda* Bleeker. Mar. Pollut. Bull..

[91] Wang Y.X., Liu M.J., Geng X.H., Zhang Y., Jia R.Q., Zhang Y.N., Wang X.X., Jiang Y. (2022). The combined effects of microplastics and the heavy metal cadmium on the marine periphytic ciliate *Euplotes vannus*. Environ. Pollut..

[92] Yu Y., Liu J., Zhu J., Lei M., Huang C., Xu H., Liu Z., Wang P. (2024). The interfacial interaction between typical microplastics and Pb2^+^ and their combined toxicity to *Chlorella pyrenoidosa*. Sci. Total Environ..

[93] Wang L., Gao Y., Jiang W., Chen J., Chen Y., Zhang X., Wang G. (2021). Microplastics with cadmium inhibit the growth of *Vallisneria natans* (Lour.) Hara rather than reduce cadmium toxicity. Chemosphere.

[94] Soliman H., Salaah S., Hamed M., Sayed A. (2023). Toxicity of co-exposure of microplastics and lead in African catfish (*Clarias gariepinus*). Front. Vet. Sci..

[95] Zhang J., Lin Z., Ai F., Du W., Yin Y., Guo H. (2024). Effect of ultraviolet aged polytetrafluoroethylene microplastics on copper bioavailability and Microcystis aeruginosa growth. Aquat. Toxicol..

[96] Sánchez-Fortún A., D’ors A., Fajardo C., Costa G., Sánchez-Fortún S. (2024). Influence of polyethylene-type microplastics on long-term exposure to heavy metals in freshwater phytoplankton. Sci. Total Environ..

[97] Sánchez-Fortún A., D’ors A., Fajardo C., Martín C., Nande M., Mengs G., Costa G., Martín M., Sánchez-Fortún S. (2022). Influence of contaminant-spiked polyethylene-type microplastics on the growth and primary production of the freshwater phytoplankton species *Scenedesmus armatus* and *Microcystis aeruginosa*. Environ. Exp. Bot..

[98] Wang S., Li Q., Huang S., Zhao W., Zheng Z. (2021). Single and combined effects of microplastics and lead on the freshwater algae *Microcystis aeruginosa*. Ecotoxicol. Environ. Saf..

[99] Rehse S., Kloas W., Zarfl C. (2018). Microplastics reduce short-term effects of environmental contaminants. Part I: Effects of bisphenol A on freshwater zooplankton are lower in presence of polyamide particles. Int. J. Environ. Res. Public Health.

[100] Le Bihanic F., Clérandeau C., Cormier B., Crebassa J.-C., Keiter S.H., Beiras R., Morin B., Bégout M.-L., Cousin X., Cachot J. (2020). Organic contaminants sorbed to microplastics affect marine medaka fish early life stages development. Mar. Pollut. Bull..

[101] Syberg K., Nielsen A., Khan F.R., Banta G.T., Palmqvist A., Jepsen P.M. (2017). Microplastic potentiates triclosan toxicity to the marine copepod *Acartia tonsa* (Dana). J. Toxicol. Environ. Health A.

[102] Gholamhosseini A., Banaee M., Zeidi A., Multisanti C.R., Faggio C. (2024). Individual and combined impact of microplastics and lead acetate on the freshwater shrimp (*Caridina fossarum*): Biochemical effects and physiological responses. J. Contam. Hydrol..

[103] Zhang S., Ding J., Razanajatovo R.M., Jiang H., Zou H., Zhu W. (2019). Interactive effects of polystyrene microplastics and roxithromycin on bioaccumulation and biochemical status in the freshwater fish red tilapia (*Oreochromis niloticus*). Sci. Total Environ..

[104] Zhu Z.-L., Wang S.-C., Zhao F.-F., Wang S.-G., Liu F.-F., Liu G.-Z. (2019). Joint toxicity of microplastics with triclosan to marine microalgae *Skeletonema costatum*. Environ. Pollut..

[105] Wang Z., Fu D., Gao L., Qi H., Su Y., Peng L. (2021). Aged microplastics decrease the bioavailability of coexisting heavy metals to microalga *Chlorella vulgaris*. Ecotoxicol. Environ. Saf..

[106] Dong Y., Gao M., Qiu W., Song Z. (2021). Effects of microplastic on arsenic accumulation in *Chlamydomonas reinhardtii* in a freshwater environment. J. Hazard. Mater..

[107] Liu Q., Wu H., Chen J., Guo B., Zhao X., Lin H., Li W., Zhao X., Lv S., Huang C. (2022). Adsorption mechanism of trace heavy metals on microplastics and simulating their effect on microalgae in river. Environ. Res..

[108] Avio C.G., Gorbi S., Milan M., Benedetti M., Fattorini D., d’Errico G., Pauletto M., Bargelloni L., Regoli F. (2015). Pollutants bioavailability and toxicological risk from microplastics to marine mussels. Environ. Pollut..

[109] Jeong C.-B., Kang H.-M., Byeon E., Kim M.-S., Ha S.Y., Kim M., Jung J.-H., Lee J.-S. (2021). Phenotypic and transcriptomic responses of the rotifer *Brachionus koreanus* by single and combined exposures to nano-sized microplastics and water-accommodated fractions of crude oil. J. Hazard. Mater..

[110] Lee Y., Yoon D.-S., Lee Y.H., Kwak J.I., An Y.-J., Lee J.-S., Park J.C. (2021). Combined exposure to microplastics and zinc produces sex-specific responses in the water flea *Daphnia magna*. J. Hazard. Mater..

[111] Li Y., Yang G., Wang J., Lu L., Li X., Zheng Y., Zhang Z., Ru S. (2022). Microplastics increase the accumulation of phenanthrene in the ovaries of marine medaka (*Oryzias melastigma*) and its transgenerational toxicity. J. Hazard. Mater..

[112] Yan W., Hamid N., Deng S., Jia P.-P., Pei D.-S. (2020). Individual and combined toxicogenetic effects of microplastics and heavy metals (Cd, Pb, and Zn) perturb gut microbiota homeostasis and gonadal development in marine medaka (*Oryzias melastigma*). J. Hazard. Mater..

[113] Santos D., Félix L., Luzio A., Parra S., Bellas J., Monteiro S.M. (2021). Single and combined acute and subchronic toxic effects of microplastics and copper in zebrafish (*Danio rerio*) early life stages. Chemosphere.

[114] Chen X., Peng L.-B., Wang D., Zhu Q.-L., Zheng J.-L. (2022). Combined effects of polystyrene microplastics and cadmium on oxidative stress, apoptosis, and GH/IGF axis in zebrafish early life stages. Sci. Total Environ..

[115] Paul-Pont I., Lacroix C., Fernández C.G., Hégaret H., Lambert C., Le Goïc N., Frère L., Cassone A.-L., Sussarellu R., Fabioux C. (2016). Exposure of marine mussels *Mytilus* spp. to polystyrene microplastics: Toxicity and influence on fluoranthene bioaccumulation. Environ. Pollut..

[116] Romdhani I., De Marco G., Cappello T., Ibala S., Zitouni N., Boughattas I., Banni M. (2022). Impact of environmental microplastics alone and mixed with benzo [a] pyrene on cellular and molecular responses of *Mytilus galloprovincialis*. J. Hazard. Mater..

[117] Pittura L., Avio C.G., Giuliani M.E., d’Errico G., Keiter S.H., Cormier B., Gorbi S., Regoli F. (2018). Microplastics as vehicles of environmental PAHs to marine organisms: Combined chemical and physical hazards to the Mediterranean mussels, *Mytilus galloprovincialis*. Front. Mar. Sci..

[118] Xie C., Li X., Chen Y., Wu X., Chen H., Zhang S., Jiang L., Pang Q., Irshad S., Guo Z. (2024). Impact of Polystyrene Microplastic Carriers on the Toxicity of Pb2^+^ towards Freshwater Planarian *Dugesia japonica*. Environ. Sci. Nano.

[119] Li Y., Wang J., Yang G., Lu L., Zheng Y., Zhang Q., Zhang X., Tian H., Wang W., Ru S. (2020). Low level of polystyrene microplastics decreases early developmental toxicity of phenanthrene on marine medaka (*Oryzias melastigma*). J. Hazard. Mater..

[120] Lu K., Qiao R., An H., Zhang Y. (2018). Influence of microplastics on the accumulation and chronic toxic effects of cadmium in zebrafish (*Danio rerio*). Chemosphere.

[121] Wang S., Xie S., Zhang C., Pan Z., Sun D., Zhou A., Xu G., Zou J. (2022). Interactions effects of nano-microplastics and heavy metals in hybrid snakehead (*Channa maculata*♀ × *Channa argus*♂*)*. Fish Shellfish Immunol..

[122] Jeong H., Lee Y.H., Sayed A.E.-D.H., Jeong C.-B., Zhou B., Lee J.-S., Byeon E. (2022). Short-and long-term single and combined effects of microplastics and chromium on the freshwater water flea *Daphnia magna*. Aquat. Toxicol..

[123] Lee J.-S., Oh Y., Park H.E., Lee J.-S., Kim H.S. (2023). Synergistic toxic mechanisms of microplastics and triclosan via multixenobiotic resistance (MXR) inhibition–mediated autophagy in the freshwater water flea *Daphnia magna*. Sci. Total Environ..

[124] Yang Z., Zhu L., Liu J., Cheng Y., Waiho K., Chen A., Wang Y. (2022). Polystyrene microplastics increase Pb bioaccumulation and health damage in the Chinese mitten crab *Eriocheir sinensis*. Sci. Total Environ..

[125] Wang S., Xie S., Wang Z., Zhang C., Pan Z., Sun D., Xu G., Zou J. (2022). Single and combined effects of microplastics and cadmium on the cadmium accumulation and biochemical and immunity of *Channa argus*. Biol. Trace Elem. Res..

[126] Zhang C., Zhang L., Li L., Mohsen M., Su F., Wang X., Lin C. (2023). RNA sequencing provides insights into the effect of dietary ingestion of microplastics and cadmium in the sea cucumber *Apostichopus japonicus*. Front. Mar. Sci..

[127] Qiao R., Lu K., Deng Y., Ren H., Zhang Y. (2019). Combined effects of polystyrene microplastics and natural organic matter on the accumulation and toxicity of copper in zebrafish. Sci. Total Environ..

